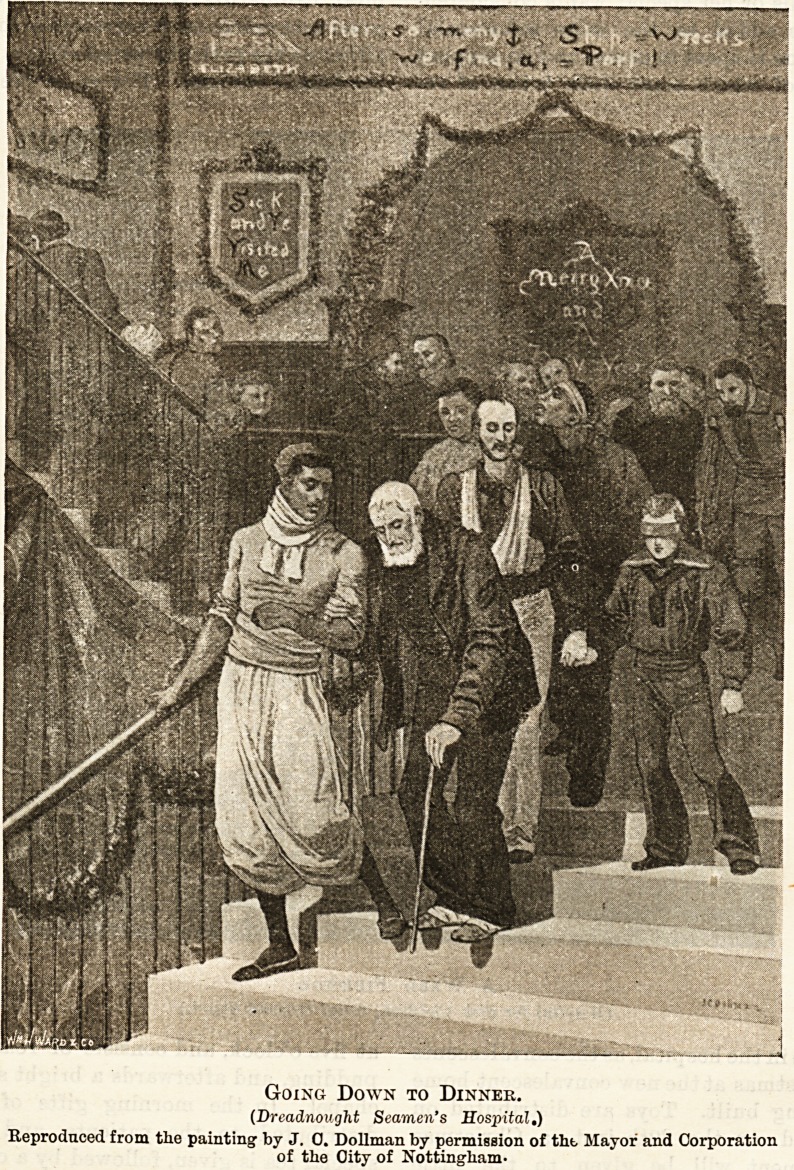# Christmas Appeal Supplement

**Published:** 1897-12-18

**Authors:** 


### CHRISTMAS APPEAL SUPPLEMENT—ILLUSTRATED.

### The End of a Jubilee.

Never, perhaps, was a year in which hospitals attracted so large a share of public attention as they have done in the one which is now drawing to a close. To a great extent this has been due to the fact that while national sentiment has marked it as a year of jubilee—a year in which to celebrate the more than Jubilee of Her Majesty's reign—the royal desire, as intimated by H.R.H. the Prince of Wales, has been that the celebration of the event should take the form of support to hospitals. Thus it has happened that a great deal of public interest has been excited in regard to these institutions; an interest which has not always shown itself in the form of a donation. Money has undoubtedly been given, but in still greater volume has criticism been poured out; and although criticism is a thing which always, in the end, does good, there can be but little question that as a result of all the talk about central boards, hospital abuse, hospital control, hospital reform, and what not, a feeling has been gradually instilled into the minds of some that there is something rotten in the management of hospitals and something doubtful in regard to their utility. This, however, is a feeling which must be removed, and we have no hesitation in saying that it is without good grounds on which to rest.

Christmas-time is a time when we should be in charity with all men, and when this charity should especially take the form of help to the feeble and the sick; and at this time in particular it seems appropriate that we should again urge the claims of the hospitals, and that we should enforce attention to the fact that, notwithstanding all the talk about abuse and about reform, and notwithstanding that there may be some basis for believing that in certain matters hospitals, like everything else, are capable of some improvement, they are in the main doing an enormous amount of good—good of a sort that cannot be done by any other means, and in a way for which no substitute has been or can be discovered.

The great central fact which has to be recognised is that the lives of the masses—the modes of life imposed upon great numbers of our fellow-citizens by the inexorable demands of what we are pleased to call modern civilisation—are such that when they fall ill it is impossible, on the one hand, for them to provide for themselves the medical attendance that is necessary; and, on the other, for them to receive proper benefit from that attendance, even if it could be had, so long as they remain amid the surroundings in which they have been taken ill — surroundings which, in far too many instances, have themselves been the real cause of the illness.

Here and there a well-to-do person may gain access to a hospital, and may cheat the subscribers; but that should not harden our hearts and close our pockets, for these cases are lost in the mass of the really poor and of the really deserving, for whom, if help is to come at all, it must come in the form of hospital treatment.

For the true understanding of the matter, concrete examples are worth much talk. Let us, then, take the experience gained quite lately by a lady, Miss Margaret Hardinge Irwin, who has recorded in the *Westminster Review* the results of her investigations into the earnings of women of various trades who work at home: "E. B. is a young woman living with her parents. She works steadily from seven in the morning until nine at night. She can very seldom make as much as 8s. a week, and often only 4s., especially with the flannelette shirts which chiefly employ her." The workers, having no organisation among themselves to fix and maintain a standard and uniform rate of wages, "are obliged to accept the reductions pressed upon them by less scrupulous or necessitous employers, until in many cases wages have sunk below the actual 'starvation level,' and have to be supplemented by public or private charities."

But if we consider the case even of those who work in factories, the very anxiety some of them show to take work home with them is enough to prove how small must be the wages earned by their ordinary work. "One woman informed me that she had 'never known a worker who did not take work home from the shop. They rise at six and work before they go in, and bring back enough at night to keep them going till twelve; they couldn't live if they didn't, and with it all they can't make more than 9s. a week.'"

Some of the tenements occupied by home workers are said to be filthy in the extreme, the very dirt of the places rendering it out of the question to attempt to treat the sick in such habitations. "Armed with a box of matches and a taper, and battling with what seem to be the almost solid smells of the place, one finally reaches the top, and on being admitted finds, perhaps, a room almost destitute of furniture, the work lying in piles upon the dirty floor or doing duty as bed-clothes for a bedridden invalid and members of the family generally." It is obvious at the very first glance that it is impossible to treat sickness amid such conditions. Such dirt, however, is not always present. Side by side with some even of the worst of these tenements, one may find "a tiny room exquisitely clean and neat, and representing a world of sacrifice and effort. We often hear it said, 'People can at least be clean; cleanliness costs nothing.' But in these city slums, where the water supply is difficult of access, and sanitary provisions of any kind are practically non-existent, cleanliness is not a cheap and easy thing, but a hardly-earned luxury, almost beyond the reach of the weary, over-driven mother, and the worn-out delicate woman." Every half-hour devoted to cleanliness may mean the loss of a meal to the worker.

Now, has not all this a bearing on what we so often hear about hospital abuse. Even if we were to grant all that is said against hospitals—a thing which we should not think of doing for a moment—that would not take away from the immensity of the real distress which hospitals alone are able to relieve. What we have to bear in mind is that home workers are in very many cases among the most thrifty, the most careful, and the most honest of the various classes into which we may divide the poor. Economically, all sorts of evil things are said against them by the rest of the working community. It is said that it is the eagerness of the home worker for work that keeps down wages, and it may be so; but at least this is to be said, that it is by dint of what the world counts to them for virtues—thrift, sobriety, honesty, and punctuality—that they are able to live as they do, and that at least the people who exercise these virtues, year in and year out, till sickness overtakes them, deserve something better than the workhouse when that dreaded event happens. Let us look at the household expenditure of a widow who has kept herself and little girl on 6s. a week, "for rent and everything": Rent, one room, 2s. a week; ¼ lb. tea, 4d.; 2 lb. sugar, 3d.; flour, 1½d.; oatmeal, 2½d.; ½ lb. margarine, 3½d.; six eggs (chipped), 3½d.; ham, 2½d.; coals, 3d.; onions, or other vegetables, 1½d.; bread, 4½d.; "kitchen," a term used to denote any little relish to make up a meal, 2d. or 3d.; making a total of about 4s. 7d. or 4s. 8d. a week.

Again, take the details given by the wife of a cobbler earning 13s. a week, with four children, the eldest 11 years, the youngest eight months: Bread, 5½d. per day; tea and sugar, 3d.; "kitchen," 4d.; milk, 2d.; butter, 3d.; coals, 1s. 2d. per week; oil, 3d. She "counts on keeping the six of them on 15s. from Saturday to Monday."

Now, we ask, Where is the elasticity in such an expenditure? Where is the reserve to stand the strain of sickness? We answer that there is no elasticity, and there is no reserve, and unless thrifty, careful, self-supporting working people like these are to be thrown into the workhouse when ill, the hospitals are an absolute necessity in the present condition of our social system.

Such details as these are dull reading, and may seem to have but slight connection with the question of the hospitals; but really they are of the very essence of the question, for they demonstrate the existence of a degree of respectable poverty which it is the first aim and intention of the hospital system to help when sickness is added to its other miseries. What is true of home workers is also true, unfortunately, of those with whom they compete. The widespread outcry against home work is but a proof of the largeness of the class of factory workers who are affected by the competition so caused; and so exceeding fine does modern competition grind the worker that we may be quite sure that the examples we have quoted above are but examples which might be multiplied, almost *ad infinitum*, from among the ordinary working population which forms the mass of the dwellers in all great cities. Let no one at this Christmas-time button up his pockets. Let no one allow himself to be deterred by vague rumours of mismanagement to salve his conscience with the idea that the call for hospitals is not urgent. Let no one be betrayed into the optimistic belief that times are fairly good, and that the number of really deserving objects is but small. Those who know, know differently; for they know that a ghastly poverty permeates our cities, that behind our gay streets, our bright shops, our well-dressed crowds, behind the light, the life, the bustle which go to make up the best known aspect of our great London, there lies a sea of dark courts and alleys, a quagmire of poverty, an untold mass of tenements, in which healthy people can live no doubt, but in which the occurrence of sickness always means the threatening of death. To the dwellers in such places the hospitals remain, and must always remain, an absolute necessity.

We, therefore, urge again, as we have urged many times already, that at Christmastide, above all times, it is good to give, and that our hospitals, above all other institutions, are the most worthy to be the recipients of our charity.

### "Well Contented."

The subject of the full-page illustration which we issue with this Christmas Supplement for 1897 has been selected with an object. That object is to bring home to the hearts of men and women of all classes one truth at least as to hospitals and the patients they contain. It is probably true that there is not a voluntary hospital in this country where a sick person when really ill would not find greater comfort in one of its beds than almost anywhere. "Well Contented" the child in our illustration undoubtedly is. Indeed, it has good cause to be so, for it is better cared for, better fed, and probably on the whole happier in that hospital cot than it has ever been in its life before. Besides, it is a resourceful child, as its expression shows, and its thoughts at the moment are far away, or, at least, far removed from the contemplation of the immediate objects by which it is surrounded. Gentle reader, study and ruminate upon this picture of ours. If you love children, let your fancy wander into Wonderland as it used to do in the long ago, and then let your heart be touched and your sympathies aroused to a sense of the privilege of helping to succour the sick, and especially the sick children, who may be fit inmates for our hospitals. The following verses by the late Robert Louis Stevenson may bring a blessing to you this Christmastide, as they have already brought happiness to others in past years:—

"When I was sick and lay abed,
I had two pillows at my head;
And all my toys beside me lay
To keep me happy all the day.

"And sometimes for an hour or so
I watched my leaden soldiers go,
With different uniforms and drills,
Among the bedclothes, through the hills;

"And sometimes sent my ships in fleets
All up and down amongst the sheets;
Or brought my trees and houses out,
And planted cities all about.

"I was the giant great and still
That sits upon the pillow hill,
And sees before him dale and plain,
The pleasant land of counterpane."

### Christmas in the Hospitals.

Christendom stands once more on the eve of the great festival of good-will. For awhile the whirl of toil ceases, and brother finds time to greet brother in mutual good fellowship, reconciliations knit together severed friendships, and messages of sympathy and help are sent forth to the sick, the suffering, and the sad.

How appalling in the larger towns is the number of the "submerged tenth" which is hidden in tenements and slums, whose daily problem is to find their daily food when chills of winter pierce their fireless homes, their scanty clothing, their hunger-weakened frames; then is disease most rife and the earning power of the miserable at its lowest. Is this really so? Ask the district nurse as she goes her rounds; ask the medical men and students as they pursue their calling. They see it always everywhere; they tax their own resources to the utmost. Is it not a fact that some district nurses have to be forbidden to take their earnings to relieve the destitution that they meet upon their rounds?

Not far from Paddington there is a band of district nurses. They have worked so long in the neighbourhood that they are well known by the poor, who hasten to them for help in cases of sickness more serious than usual. A mother one day sent word that her baby was ill, and asked that a nurse should see to the child for her, as she had work that she could not afford to lose, The nurse accordingly called; this is what she found—an absolutely bare room, no chair, no table, no pot, nor pan, nor kettle; a sick, naked baby on a heap of waste straw, covered with a bit of dirty blanket lent by a neighbour almost as poor as the object of her charity. The child was very ill and very dirty. The nurse, with the help of a neighbour's boy, made a fire with some torn posters, an old box, and some bits of wood collected in the street. Another neighbour lent her kettle, and when the water was hot a cracked pie-dish was discovered, which served as a wash-basin, and the Samaritan fund supplied a garment. It is not difficult to imagine the impossibilty of keeping Christmas in such a home, for poverty is no respecter of seasons, and, while the joy-bells of Christmas call all to the feast, the guests of the highways and bye-ways, those whom the Master bade his servants compel to come in, are often left desolate. How grievous this flotsam and jetsam of human wreckage. Some, doubtless, have themselves to blame for their sorry plight, but the majority suffer with and for the sins of others. How can a child grow to vigorous stature unless it be fed? How can it learn to do well unless it be taught?

The greatest refuges of the destitute are our hospitals, and it is only necessary to visit the incurable wards of one or another in order to see what can be done with and for those poor folk, and that those who cannot

The Children's Christmas Tree.
*(Victoria Ward, St. Thomas's Hospital.)*

pay such a visit may understand how great a work is being done, and how much labour is being expended in friendly effort to bring to the sick and suffering some inkling of the joy of Christmas, and some reminder that they are not forgotten, we have visited all the hospitals ourselves, and give to our readers the following outline of what is being done in each.

Each hospital lends itself to some special festivity. At St. Thomas's, and some others, for instance, the probationers and nurses in the training school form themselves into a choir; and on Christmas morning, as they go down the corridors to their work, sing carols. As regards decorations there are two camps. Some medical men and hospital managers perceive the omnipresent microbe in the harbourage of evergreens; they declare that the labour is too fatiguing for nurses and for patients. Their opponents say that the microbe can be subdued by good management, and that the nurses and patients are all the better for having something pleasant to think about. St. Thomas's belongs to the last-named camp, as the following description of the Victoria Ward for children bears witness. This is a handsome ward to begin with, and the little cots around the sides are curtained with a deep yellow cretonne. The three fires are in the middle of the wards, and the chimneys, being round, have the appearance of pillars supporting the roof. Art muslin of a deep yellow and pale ivory colour will drape the walls, and a fairy lamp with a yellow shade will be hung from each festoon. At the entrance of the ward, two angels, suspended from the roof, will hold a scroll bearing the wish "A Happy Christmas," and immediately underneath a beautiful snow scene will be arranged. Beyond will be seen the glittering tree, the thick green wreaths, and golden Chinese lanthorns. Every little one will wear a new frock of pale blue flannel with white facings on Christmas Day, and it would be difficult to imagine a more fairy-like scene. On New Year's Eve a real Father Christmas will attend to distribute the gifts. The idea of the angels is especially charming, and will delight the imagination of the babies. For the men the fare of roast beef, plum pudding, and ale for dinner, with tobacco, is satisfactory to an Englishman's appetite and taste. Friends to tea, and visits by the medical staff and nurses and choir, make a fitting ending to a joyous day. On Boxing Day the matron will give an "At Home," and afterwards carols will be sung in the wards. The entertainment given to the nurses by the medical staff will take place about the middle of January.

Christmas is always a time of great rejoicing at Guy's Hospital. It is most extensively and beautifully decorated (though a prudent matron dismantles the walls with the new year). The members of the resident medical staff carve the beef, and the nurses wait upon the patients, and afterwards both eat their own dinner in the ward. Neither patients nor their attendants would willingly have any change in this; in fact, the wards give dinner parties, and none would be absent on any account. During Christmas week an entertainment will be given every evening at one ward or another, subject, of course, to medical permission. The novel feature this year, however, is the students' tea to 400 children belonging to the out-patient department. One of the students felt the contrast between the lot of the in-patient and those of the out-patient children so keenly that he set to work, and, with great success, collected the funds for a tea not only for all the actual out-patient children, but for those children whose relatives are on the out-patient roll. The district nurses working in connection with the hospital are fully cognisant of the circumstances of these little ones, therefore the feast is spread in this case only for those who would otherwise have had none. There will be gifts, too, on this occasion, each child being given a loaf of bread and a packet of tea.

A Christmas Interior.
(*Brompton Hospital for Consumption.*)

At Westminster Hospital every patient wakes up to find a couple of gifts on his or her pillow. There is a charming service in the chapel, which is decorated with evergreens and baskets of flowers. The memory of those of the nursing staff who have died on duty is kept fresh on this day, and the tablets which commemorate them are polished especially bright. After service comes the dinner of turkey and fowl and pudding. The nurses dine in the board-room, which is, of course, gay with evergreens and flowers. Muffins and cakes make their appearance at tea time, and then the patients are allowed to visit one another's wards, a privilege greatly appreciated. Two wards are always especially interesting—the children's ward and that for incurables. In the former Sister Margaret has reigned for upwards of ten years, and has the art of mothering and keeping babies happy in a wonderful degree. She has at her command cupboards full of treasures to infant minds, where the "orange daddy brought" is to be found at the right moment, and dolls and toys are put away to come out fresh another day. The incurable ward is very prettily decorated. The children's Christmas tree owes its fairies to busy "Florrie's" crippled fingers, and the nice old invalid ladies have knitted no end of woollen cuffs and babies' socks for the Christmas gifts. They are well knitted, too, neither too tight nor too loose, and every stitch even. Nor must the useful needlework books, the work of another inmate, be forgotten; they are just the things for pocket or work-basket. A little carol-singing ends a most enjoyable day, and a patient has been heard to say, "Well, I've never thought we could be so happy without a drop of drink."

Not far away, but south of the Thames, in that most desolate district near the Borough, lies the Royal Hospital for Children and Women, Waterloo Bridge Road, S.E. The Christmas treat for the children will be given one day during Christmas week, and the plan arranged is one full of pleasure for the little ones for whom it is intended. A capital tea will be provided at four o'clock, at five the Christmas tree will yield its magic gifts, and at six either a Punch and Judy or a magic lantern show will be exhibited, leaving time for the happily-excited but weary babies to be in bed by seven.

The next group of hospitals is to be seen at Brompton. First, of course, in size and importance is the Hospital for Consumption.

tokens of remembrance for everyone. Gifts of useful articles are made to the patients, and the special needs of each patient are carefully considered in making a selection from the tree. The whole of the staff are presented with free tickets for a theatre or other public amusement.

The Ladies' Committee is the fairy godmother of the Chelsea Hospital for Women, and the nurses vie one with another in lavishly decorating the wards. Flowers, fairy lamps, and flags are all pressed into service to produce a good effect. On Christmas Day itself, the patients feast on turkey, plum pudding, mince pies, candied, and other fruits. They may also invite a friend to tea, during which the nurses sing carols and hymns. The dinner for the nurses is daintily spread in the board-room, as much care being

A Circular Ward.
*(Great Northern Central Hospital.)*

Christmas reveals the many personal friends of the patients in Brompton Consumption Hospital. Some give the turkeys for dinner, others the fruit and crackers for dessert, and others, again, the wine. At the dinner-hour, when the medical officers and chaplain preside, the galleries present a very striking appearance. The nurses preside in the afternoon over tea-tables laden with cakes, and all other good things. In the evening there is music, and the men smoke. The chapel is much frequented on Christmas Day, services being held at six, eight, and eleven a.m. The nurses and probationers sing carols in the galleries. On the 30th inst. a magnificent Christmas tree will be given by Nurse Heddy, and her friends, on which there is to be bestowed upon the decorations and serving of the meal as on that in the nurse's own home. Afterwards a social evening with games and music is spent, and greatly appreciated. The Ladies' Committee provide extras and gifts all round. Early in January they also give a tea; after which a tree, the gift of Dr. and Mrs. Fenton, is the great attraction. Everyone, both nurses and patients, receives a really handsome present, the latter generally of warm clothing.

The Victoria Hospital for Children at Chelsea provides a very happy Christmas for its little patients, of which there are generally 65. Turkey and plum-pudding appear at the dinner hour, and cakes and other good things at tea. The annual tree, which is given by

Mrs. Guest, in the Cadogan Ward, blossoms early in January, so the children have another treat in store, at which H.R.H. the Princess Louise, the patron, is generally present.

Several important hospitals lie fairly close together in the North of London. Take first the largest, The Hospital for Sick Children in Great Ormond Street. As a rule there are about 155 beds occupied, and there are a very large number of out-patients. Although this hospital was only rebuilt in 1875, the adjoining property, the proposed purchase of which was lately announced, is already needed for the accommodation of the nursing staff. Our plate, "Well Contented," will give to our readers some idea of the state of beatitude enjoyed by the baby invalids at Christmas in this hospital. The picture may also be taken as a sample of what Christmas is made for the sick little ones of London by the hospitals generally. The particular form of rejoicing this year for 200 little guests belonging to the out-patient department is first a splendid tea, then a variety entertainment followed by a distribution of gifts from a noble tree. It takes place on the 28th inst., and on the 29th the in-patients will have their treat. Every inmate will then be allowed to invite two guests to tea, in itself a source of delight, for children are nothing if not sociable. The wards will be illuminated with fairy lamps, and there will be a toy-laden tree in each ward. The entertainment will conclude with music and singing.

The adornment of the Royal Free Hospital is secured by the gift of a palm to each ward for Christmas. These are bought in Ghent, being hardier than those from English hot-houses. The wards are also decorated with evergreens, lanterns, and fairy lamps, with which very effective results are obtained. As each patient has the privilege of inviting a friend to tea, and all the children patients of the past year have also invitations, the number of willing and welcome guests is large. The Christmas dinner consists of roast beef and plum pudding. The children's stockings are hung up and mysteriously filled by Santa Claus, whilst the men folk are permitted to smoke at certain hours, and are provided with a present of tobacco. On December 15th and 16th the students give dramatic entertainments to their friends, patients, and nurses; and the children are, of course, delighted with their Christmas tree. It is usual, here as elsewhere, for everyone to receive some little memento of the occasion. The extra expenses are met by a subscription amongst the staff, and by the kindness of various friends.

The arrangements for Christmas at the National Hospital for the Paralysed and Epileptic, both at Queen's Square and at the Finchley Convalescent branch, are very complete. The chapel and wards will be decorated, a choir of nurses and sisters will sing carols early in the morning, and at breakfast each patient will receive a card and gift. A service will be held in the chapel, after which will follow a dinner of roast beef, turkeys, and plum pudding. A series of entertainments will be given at intervals throughout the day by the lady superintendent, the resident medical officer, and the steward. The round of merry-making is kept up until twelfth-night. There will be a Christmas tree, ward teas, concerts, and other entertainments, which will doubtless prove a source of great delight to all taking part in them, both performers and onlookers.

The Great Northern Central Hospital is one of the most beautiful, as it is one of the poorest, of London hospitals. On an average 87 beds are occupied, and our illustration of the circular ward gives an excellent idea of the airy, roomy environment of its sick. The Christmas dinner of turkey, plum pudding, and other delicacies is the gift of the medical staff; and the tea, with its unusual dainties, that of the Ladies' Association. The Association also presents each patient with a parcel, in which warm clothing is to be found for the elders, and toys for the children. All are amused in the evening by carol singing. Miss Middleton, a member of the Association, undertakes to arrange a musical and dramatic entertainment for January 14th, when a stage will be fitted up in one of the wards.

The London Temperance Hospital will be the scene of a couple of excellent musical entertainments this year, the first of which takes place on Christmas night, when Madame Fortescue and her friends provide the music. On that evening each inmate is allowed to invite a friend, and refreshments will be served for all. The second is on New Year's Eve, when Miss Lucie Johnstone will give a concert, and Canon Fleming some recitations, after which the Christmas tree will be stripped and the useful and pretty gifts distributed. The patients' fare on Christmas Day is turkey and plum pudding.

The rest of London hospitals mostly lie as a chain through the middle of London. Beginning at the far east there is the Poplar Hospital for Accidents, where the first event on Christmas Day is the distribution of a parcel to each patient, containing no less than three articles of warm clothing and a Christmas card; whilst the children have the perennial joy of a stocking filled by Santa Claus. The morning brings the chaplain and church choir, which visits the wards and sings carols; and then comes dinner, of roast chicken and a blazing plum pudding, followed by dessert, pipes, and tobacco. At four there is tea with cake, and later a conjurer makes his appearance, and his performance is accompanied by music and singing. Early in the New Year the students of the London Hospital kindly give a dramatic entertainment; and, at the same time, a Christmas tree is provided for the children.

Christmas Day is welcomed in the busy London Hospital by the nurses and probationers marching in procession through the wards singing carols. At ten a second party of choral singers, brought by Miss Caird from South Hampstead, spend the intervening hours until dinner amusing the patients. Early in the morning also Christmas letters and cards are dispersed, to be followed later by a personal visit from "Father Christmas," who distributes the gifts, held in readiness by the sisters, to the delighted recipients. Roast beef and plum pudding is the staple fare on Christmas Day, but some wards are lucky enough to have turkeys sent them, and in some the feast is all the more delightful because the patients' own doctor helps the pudding. After an excellent tea a variety of suitable entertainments is given, the men having the privilege of smoking all day long in their own apartments.

"WELL CONTENTED"

*By Permission of the Secretary of the Hospital for Sick Children, Great Ormond Street.*

To the North of the London Hospital is the very beautiful CITY OF LONDON HOSPITAL FOR DISEASES OF THE CHEST, Victoria Park. The long corridors are prettily decorated for the festival, and there is much singing of carols and hymns. The library and smoking-rooms are, however, wisely kept in silence, so that all who prefer quiet can enjoy it. The chapel is beautifully decorated, and short services are held at half-past seven a.m. and after the medical officer's visit is over. Dinner is laid in the day-rooms, and the officers and committee preside over the beef and chicken, plum puddings and mince pies, and the nurses are careful that each inmate should have his or her special wants considered. Ale and dessert are added to the more substantial viands. The afternoon is spent in games and in dipping into bran pies, out of which some little token of remembrance and goodwill is generally forthcoming for everyone. Cake and jams are served at tea, and little impromptu entertainments that suggest themselves to any of the staff are carried out, whilst carol singing fills up any odd half-hour. A series of entertainments, extending well into the New Year, help and prolong a bright Christmas.

By the riverside at Shadwell is the EAST LONDON HOSPITAL FOR CHILDREN, and much care is spent on providing amusement, as well as good fare for the little folk within it. Next year, most probably, none but those really ill will be in the hospital, as the convalescents will be keeping Christmas at the new convalescent home at Bognor, now being built. Toys are distributed on Christmas Day, and on the 29th inst. a Christmas tree and entertainment will be given to the little patients.

On the opposite side of the river lies the "DREADNOUGHT" SEAMEN'S HOSPITAL. The "Hearts of Oak" of all nations, driven disabled into this most useful port, sit down side by side—such as are able—to keep the feast right cheerily; after which they will enjoy that luxury so dear to sailor men—their pipes and 'baccy. On January 5th there is a Christmas tree for the smaller "craft," and children who have been inmates in the hospital during the year, are made welcome. The nurses, too, are not forgotten, but their treat comes later.

Next rises the venerable pile of opulent ST. BARTHOLOMEW'S, and however happy Christmas may be here, it is kept very quietly. Decorations although discouraged, are not forbidden, consequently there is some adornment in most of the wards.

UNIVERSITY COLLEGE HOSPITAL, just free from a severe epidemic amongst its nurses, with a treasury stated to be so empty that 50 beds will be closed, and with all the discomfort of the bricks and mortar that will four years hence be Sir Blundell Maple's magnificent hospital, can hardly keep Christmas this year with any great magnificence. Nevertheless, there is much to enjoy, although the patients' usual visitors are not admitted for prudential reasons. The roast beef and plum pudding are carved and served in the wards, at which the resident medical staff are present, much to the gratification of the patients. Tea is also a delightful meal, and the patients will enjoy two such treats this year, since as Christmas Day falls on a Saturday, the festival will be kept earlier in the week, and the day itself will be only marked by an extra evening meal.

A WARD FIRESIDE
*(Hospital for Sick Children, Great Ormond Street.)*

The celebrations of the feast at KING'S COLLEGE HOSPITAL are in every way worthy of the day, and the decorations take the form of a profusion of plants and cut flowers. Dinner is served at five o'clock, and consists of roast turkey and plum pudding, and afterwards a bright service is held in the chapel. In the morning gifts of warm clothing are distributed to the patients, and on Boxing Day a special tea is given, followed by a concert lasting about twenty minutes in each ward.

Christmas is kept right royally from morning until night at the MIDDLESEX HOSPITAL. The nurses find gifts upon their plates at breakfast, and the patients substantial ones upon their beds. At both dinner and tea seasonable dainties are plentiful. A handsome tree, lavishly decorated and heavy with keepsakes, stands in the board-room. Bran pies will make their appearance in the wards, so that all who cannot be present at the more lively proceedings shall partake in the pleasurable surprise of putting "in a thumb and pulling out a plum," after the style of Tommy Tucker. A gaily-dressed tree adorns the boys' ward for the whole of Christmas week, and then its glories are divided amongst the inmates at the New Year. Even the babies are not forgotten, and a special tree of their own is set up in their ward.

Queen Charlotte's Hospital suffers from two great drawbacks in the keeping of Christmas. The patients are in the wards so short a time, and set up "temperatures" on such a slight occasion that very great care has to be taken to prevent undue excitement. Nevertheless, on that day the usual boiled mutton and rice gives way to turkey, and in the morning some gift of warm clothing is generally found beside the patient's pillow. The matron and nurses have a very warm corner in their hearts for the wee babies that enter this cold world at Christmas time, and when it can be managed each one is provided with a complete outfit. Each member of the permanent staff is presented later with a ticket for whatever play she may select at one of the theatres.

Going Down to Dinner.
(*Dreadnought Seamen's Hospital.*)
Reproduced from the painting by J. C. Dollman by permission of the Mayor and Corporation of the City of Nottingham.

Everyone in St. Mary's Hospital endeavours to make the patients as happy as possible on Christmas Day, and much time is devoted by the nurses and students to decorating, giving entertainments, singing carols, and joining in various games inaugurated for the pleasure of those sufficiently well to enjoy such things. In order that those who are too ill to be disturbed may have the quiet essential to them, the convalescent patients are invited into other wards where there is no case that could possibly suffer by the various amusements. On Christmas morning a gift is presented to each, the greater number being provided by the St. Mary's Aid Society. On the 29th inst. there is to be a Christmas tree for children. On January 6th an entertainment is to be given to the in-patients, and on the 7th another is provided for the amusement of the nursing and medical staffs and their friends, both taking place in the board-room.

Decorations are not yet banished from St. George's Hospital, and many charming effects are produced by the combined efforts of sisters and nurses. In the afternoon the lady visitors who kindly take an interest in the hospital come, and personally distribute Christmas gifts to all the patients. On the 31st inst., Devant will give an exhibition of his clever animated photographs in the board-room, at which we may be sure none will be absent of those who can possibly get leave to come.

Christmas is a happy time at the Samaritan Free Hospital for Women. Early in the morning Christmas letters and cards are distributed, at noon the patients' dinner of turkey and plum pudding is served, and at one the nurses dine with the matron at a prettily-decorated and appointed table, whilst the servants partake of the same good cheer at two. Each inmate is permitted to keep one of the two visitors allowed to tea. This meal is substantial, and served on tables nicely decorated and arranged. The lady friends and visitors distribute useful and acceptable gifts, nad the nurses amuse the patients with songs and carols. On Boxing Day a concert is given in a ward cleared for the purpose.

On the outermost edge of the western borders of the metropolis is the West London Hospital. The district to which it ministers is very poor and very thickly populated, and patients have to be turned away constantly; yet a little more money would throw open 80 beds in the newly-built wing. There is little time to be spent on Christmas celebrations; nevertheless, the wards are gay with plenty of plants and cut flowers, and everyone shares in the distribution of warm clothing, whilst toys fall to the children's share.

### SWEET CHARITY'S GUIDE TO CHRISTMAS GIVERS.

### GENERAL HOSPITALS.

**Charing Cross Hospital,** Agar Street, West Strand, W.C.—This institution is situated in the midst of some of the most crowded thoroughfares in the metropolis, and has, therefore, to provide for a greater number of accidents than probably any other hospital of its size. All such cases are immediately admitted without delay or difficulty. Towards the special appeal being made for £100,000 to place the hospital on a sound financial basis, and to provide accommodation for the largely-increased work thrown upon the hospital nowadays, about £35,000 has so far been received. £5,000 is required to enable the hospital to close the year without incurring any fresh liability. Secretary, Mr. A. E. Reade.

**Great Northern Central Hospital,** Holloway Road, N.—Letters of recommendation are not required at this hospital. Over 1,400 in-patients are treated annually. The hospital is unendowed, and the reliable income is quite inadequate to meet the necessary expenditure—over £8,000 being required annually from voluntary sources. Two wards containing 46 beds are still unopened for want of funds. Annual subscriptions and donations may be sent to the Secretary, Mr. Lewis H. Glenton-Kerr.

**Guy's Hospital,** London Bridge, S.E.—Guy's is too old a friend of the public to need much to be said in its favour; but so long as it has closed wards and is crippled by poverty it is a reproach to the metropolis. One ward with 43 beds is about to be reopened by the hospital authorities, but there still remain two wards, each containing 57 beds, which cannot be opened unless further funds are forthcoming. The net income of the hospital derived from its landed estates remains at £20,000 per annum less than it was before the agricultural depression, with no present prospect of improvement. Treasurer, Mr. H. Cosmo Bonsor. Superintendent, Dr. Perry.

**Hampstead Hospital,** Parliament Hill Road, N.W.—The number of in-patients in 1896 was 301, besides 164 minor accidents and casualties. Of these 211 were free cases. There were also 1,886 attendances in the out-patient department. There is a heavy mortgage on the building which the council are most anxious to reduce. Sec., Mr. R. A. Owthwaite.

**Italian Hospital,** Queen Square, Bloomsbury, W.C.—The present building is quite inadequate, and most unsuitable for the work which the hospital now does, and the committee are therefore appealing for funds to rebuild. A gentleman, by whose benevolence the work of the hospital has mainly been carried on since its establishment, has given the freehold of an adjoining house to enlarge the site for the new hospital which it is hoped shortly to erect. Funds for this purpose are appealed for. Secretary, M. G. Ferrari.

**King's College Hospital,** Portugal Street, Lincoln's Inn, W.C.—2,703 in and 25,062 out-patients were treated in 1896, in addition to 710 poor married women attended during confinement in their own homes. Warden, Rev. N. Bromley.

**London Homœopathic Hospital,** Great Ormond street, W.C.—The number of in-patients last year was 1,031, and the number of out-patients 14,500. The committee are at the present time working to provide funds for the erection of the additions to the Nursing Home, and to secure an increased income sufficient to meet the increased expenditure caused by the greater work now thrown on the hospital. Treasurer, Viscount Emlyn, 7, Princes Gardens, S.W. Secretary-Superintendent, Mr. G. A. Cross.

**London Hospital,** Whitechapel Road, E.—This is the largest voluntary hospital in this country. From this fact, and from the zealous manner in which the work is carried on, we make no doubt that the public will give largely to its funds, in testimony of their appreciation of the enormous value of what is being done there for the poor of East London. It is in serious want of funds, as the committee depend on voluntary contributions for £40,000 a year to enable them to maintain the 650 beds which are daily occupied by urgent cases. House Governor and Secretary, Mr. G. Q. Roberts, M.A.

**Metropolitan Hospital,** Kingsland Road, N.E.—The need for this hospital in the poor and densely populated districts in the midst of which it is situated, is shown by the fact that both the in and out patients have been steadily on the increase for several years past, the numbers treated last year being, in-patients, 1,013; and out-patients 26,768, the attendances of the latter numbering 87,295. There is a debt at the present time of over £7,000 incurred during the past four years. The charity is much crippled by lack of funds, and several beds have had to be closed, leaving only 70 now available for in-patients, although there is accommodation for 160. Secretary, Mr. C. H. Byers.

**Middlesex Hospital,** Mortimer Street, W.—The in-patients for last year numbered 3,598, and the out-patients 44,484; the income from all sources, including legacies, was £27,821, and the expenditure, £30,578; deficiency, £2,756. The cancer wards are a distinguishing feature of the hospital, and their extra nursing, costly treatment, and unlimited dietary add largely to the expenses of the hospital. Towards the extension of this department by removing it from the main body of the hospital and building a new wing, £5,000 is still needed. Secretary-Superintendent, Mr. F. Clare Melhado.

**North-West London Hospital,** Kentish Town Road, N.W., was founded in 1878, and is the only institution of the kind in the north-west district. It has 53 beds, and last year there were 578 in-patients and 17,800 out-patients. Secretary, Mr. Alfred Craske.

**Poplar Hospital for Accidents,** Blackwall, E.—The year 1897 has again seen improvements at this hospital. The isolation block has been opened, a new operating theatre built, and the receiving-room has been doubled in size. The work of late years has greatly increased, and consequently further subscriptions and donations are very necessary. Chairman, the Hon. Sydney Holland. Secretary, Lieut.-Colonel Feneran.

**Royal Free Hospital,** Gray's Inn Road, W.C.—Having no endowment, this hospital is entirely dependent for support on the subscriptions of its governors and the voluntary donations and bequests of its friends. It admits into its wards over 2,000 poor sick persons annually, besides administering advice and medicine to more than 25,000 out-patients who resort to it, not only from the crowded courts and alleys in its immediate neighbourhood, but from all parts of London and the suburban districts. The relief thus afforded is effected at a cost of about £11,000 per annum. Secretary, Mr. Conrad W. Thies.

**St. George's Hospital,** Hyde Park Corner, S.W.—This institution contains 351 beds, and during 1896 treated 3,873 in-patients, 13,601 out-patients, 12,936 casualties, and 455 maternity cases. Secretary and Superintendent, Mr. C. L. Todd.

**St. Mary's Hospital,** Paddington, W.—Very heavy work is thrown upon this institution owing to the large area of scattered poor which it has to assist. It is not so well supported as it should be, in spite of, or perhaps because of, its many wealthy neighbours. The board of management, therefore, urgently appeal for further support. The special wants of the hospital are: (1) New annual subscriptions; (2) donations for the endowment of beds and cots; and (3) £65 in new annual subscriptions to the Maternity Fund to defray the cost of the maternity nurse. The hospital is free, and no urgent case is refused admission. Secretary, Mr. Thomas Ryan.

**St. Thomas's Hospital,** Westminster Bridge Road, S.E.—Three wards, containing 90 beds (two of which are at present used for paying patients), have since 1883, been closed to the poor through want of funds. Seeing that in no part of the metropolis is the hospital accommodation so meagre as it is on the south side of the Thames, the district in which the hospital is situated, the want of these beds in which some 1,300 poor patients might be treated, is sorely felt. The population of the district is increasing at the rate of about 24,000 annually, and yet there are fewer beds available now than in 1871. A Bed Endowment Fund has recently been established, to which contributions of £1,000 are invited. Treasurer, Mr. T. Wainwright.

**Seamen's Hospital Society, "Dreadnought,"** Greenwich, S.E.—During 1896 representatives of over 50 different nationalities were treated in the various establishments of the Society. An effort is now being made by the Committee to enlarge the branch hospital in the Royal Albert Docks, the accommodation there being inadequate to meet the requirements of that busy centre of shipping. Secretary, Mr. P. Michelli.

**University College Hospital,** Gower Street, W.—50 beds were in November last closed because sufficient funds were not, in spite of a special appeal made by the committee, forthcoming to keep them open. The present indebtedness exceeds £13,000. Secretary, Mr. Newton H. Nixon.

**Westminster Hospital,** Broad Sanctuary, S.W.—Out of an expenditure of £15,000, less than £3,000 is assured to this charity, so that about £12,000 has to be made up each year in subscriptions, donations, and legacies. The work of the hospital in 1896 included the treatment of 2,696 in-patients, 23,077 out-patients and casualties, and 287 lying-in women. The hospital does an immense service to the country by training a large number of excellent nurses. Secretary, Mr. Sidney M. Quennell.

### PROVINCIAL HOSPITALS.

**The Birmingham General Hospital.**—The new building of this hospital was opened by H.R.H. Princess Christian, on behalf of H.M. the Queen, on July 7th, 1897, and will contain 340 beds. To meet the increased accommodation provided a larger income will be necessary, and an earnest appeal is made for new and increased annual subscriptions. House Governor, Mr. Howard J. Collins.

**Bournemouth National Sanatorium for Consumption and Diseases of the Chest.**—Patients who are convalescent, those who require further medical treatment and change of air, and those suffering from an incipient form of the disease are cases for the relief of which this institution was founded. There is accommodation for 31 men and 31 women, and 300 patients from all parts of the Kingdom are treated annually. The Committee earnestly solicit liberal support as, with the exception of a small dividend, the sanatorium is entirely dependent on voluntary contributions. Secretary, Mr. G. Lowe Riddett.

### SPECIAL HOSPITALS.

### CONSUMPTION.

**Brompton Hospital for Consumption and Diseases of the Chest,** S.W.—This hospital is well known as one where patients are well cared for, and every effort is made to brighten their clouded lives. Of late years many subscriptions to the hospital have been withdrawn through failure of income and other causes; the committee are most anxious that the places of those who formerly subscribed should be filled up by other kind friends. The committee also ask for assistance to enable them to erect a nurses' house on ground in rear of the hospital, immediate steps being necessary to increase the present inadequate accommodation for the nursing staff. Funds are also asked for towards the annual Christmas tree. Secretary, Mr. W. H. Theobald.

**City of London Hospital for Diseases of the Chest,** Victoria Park, E.—Situated in the East of London, where the diseases it treats are so common, about 1,000 in-patients and 15,000 out-patients are treated yearly. 120 beds have recently been closed, leaving but 44 available for patients. Assistance is much required to repay loan from bankers to meet current expenditure. Donations or annual subscriptions will be thankfully received. Secretary, Mr. Henry T. Dudley Rider.

**North London Hospital for Consumption,** Mount Vernon, Hampstead, N.W., and Fitzroy Square, W.—This institution was thoroughly reorganised and remodelled some years ago, and has now become one of the most useful of its class. There is room in the hospital for 80 beds, but the present income does not admit of more than 60 being opened. Hon. Secretary, Mr. L. F. Hill, M.A.

**Royal Hospital for Diseases of the Chest,** City Road, E.C.—The expenditure at this hospital exceeds £8,000, towards which there is an annual subscription list of some £2,250, and dividends amounting to about £120. Two additional wards have recently been opened, and funds are urgently needed. Donations will be gratefully acknowledged by the Secretary, Mr. John Harrold.

**Royal National Hospital for Consumption and Diseases of the Chest,** Ventnor, Isle of Wight, provides accommodation for 134 patients (83 men and 51 women). As the expenses exceed the assured income by £4,000, the committee appeal for additional annual subscriptions and donations. Secretary, Mr. Ernest Morgan, London office, 34, Craven Street, Charing Cross, S.W.

### LYING-IN.

**City of London Lying-in Hospital,** City Road, E.C.—Hundreds of poor women were safely delivered either in the hospital or at their own homes during 1896. Funds are needed to carry on the general work of the hospital. Secretary, Mr. R. A. Owthwaite.

**Queen Charlotte's Lying-in Hospital,** Marylebone Road, N.W.—This hospital received 1,151 patients in its wards last year, and in addition attended 1122 patients at their own homes. To meet the great pressure on the accommodation of the hospital an extension is being carried out, and a new nurses' home will shortly be commenced. For these works upwards of £12,000 will be needed. Donations to the building fund, as well as for general maintenance, are earnestly solicited. Secretary, Mr. Arthur Watts.

### EPILEPSY AND PARALYSIS.

**Hospital for Epilepsy and Paralysis,** Regent's Park, N.W.—The last annual report again recorded a diminished income from all voluntary sources except legacies. Secretary, Mr. H. Howgrave Graham.

**National Hospital for the Paralysed and Epileptic** (Albany Memorial), Queen Square, W.C.—The total number of beds provided at this hospital and its Finchley branch is 200, but the accommodation is still painfully insufficient to meet the requirements, as in a large majority of cases the patients are unsuited to general hospitals. The attendances of out-patients are upwards of 33,000 yearly. More than 1,500 different cities, towns, and villages have sent in patients. Besides the hospital for treatment, there is a pension fund for the incurables. The annual expenditure is nearly £16,000, of which from £9,000 to £10,000 must be raised in benefactions. Director, Mr. B. Burford Rawlings.

**West-end Hospital for Paralysis, Epilepsy, &c.,** 73, Welbeck Street, W.—A special appeal is now being made to enable the committee of this institution to pay off a debt of £6,000 incurred in rebuilding. Treasurer, Mr. H. Alexander Dowell.

CHILDREN.

**Alexandra Hospital for Children with Hip Disease,** Queen Square, Bloomsbury, W.C.—The necessity for this hospital for hip disease may best be shown by the fact that nearly three-fourths of its patients come from other London hospitals, the reason being that no general hospital can retain a patient for two or three years as is not infrequently done in this hospital. The hospital is absolutely unendowed, and depends solely upon voluntary contributions. Additional help is needed, as two wards are now closed, and though the hospital is not at present in debt, £400 is required to meet the Christmas bills. Secretary, Mr. Stanley Smith.

**East London Hospital for Children and Dispensary for Women,** Shadwell, E.—If institutions such as St. George's and St. Mary's Hospitals suffer from absence of adequate support, it will readily be understood that the struggle for existence is much greater in such a neighbourhood as Shadwell, and it is for this reason that the committee of the East London Hospital for Children appeal to the more wealthy classes for funds to enable them to help those who are unable to help themselves. Secretary, Mr. Thomas Hayes.

**North-Eastern Hospital for Children,** Hackney Road, Shoreditch.—This hospital, placed in the midst of one of the most crowded districts of East London, and with a population of nearly half a million mainly dependent upon it, is too small for the work it is called upon to do, and it is sadly in need of the enlargement, for which the committee are now trying to raise funds. Secretary, Mr. T. Glenton-Kerr. City office, 27, Clement's Lane, E.C.

**The Hospital for Sick Children,** Great Ormond Street, W.C.—An appeal is just being issued for £30,000, in order to enable this institution to purchase the adjoining premises, on which stand the Hospital and Convent of St. John and St. Elizabeth, a step forced upon the committee, owing to the proposal to build a new hospital on this site, which would interfere seriously with the light and air of the children's hospital, and with the working of the out-patients' department. Secretary, Mr. Adrian Hope.

**Victoria Hospital for Children,** Queen's Road, Chelsea, S.W. (and Victoria Convalescent Home, Broadstairs). —This unendowed hospital seeks further subscriptions to help it to carry on its work. The expenditure reaches £7,000 to £8,000 a year, and this has to be raised entirely by voluntary subscriptions. Secretary, Mr. A. C. Skinner.

WOMEN.

**Chelsea Hospital for Women,** Fulham Road, S.W. —This hospital has 52 beds, and treats respectable poor women and ladies in reduced circumstances. Contributing in-patients pay 10s. 6d. to 42s. a week, according to their means. The hospital is entirely without endowment or reserve funds, and greatly needs legacies and new annual subscriptions. Secretary, Mr. Herbert H. Jennings.

**Grosvenor Hospital for Women and Children,** Vincent Square, Westminster, S.W.—This hospital, hitherto inadequate and ill-contrived, has been recently reconstructed with modern improvements. Increased support is needed. Secretary, Mr. W. Aston-Lewis.

**New Hospital for Women,** 144, Euston Road, N.W. —Only a want of funds prevents the committee from providing the beds necessary to treat the numerous cases waiting for admission to this hospital. Each bed costs about £75 a year, and an appeal is made to generous friends to come to the aid of the committee by endowing beds with an amount that would produce this annual sum. Secretary, Miss M. M. Bagster.

**Royal Hospital for Children and Women,** Waterloo Bridge Road, S.E.—528 in-patients and 7,811 out-patients were treated during 1896. The income amounted to £3,152 and the ordinary expenditure to £4,027. Secretary, Mr. Thos. S. Conisbee.

**Samaritan Free Hospital for Women and Children,** Marylebone Road, N.W.—Some £7,000 per annum is required to maintain this hospital, of which only £1,750 can be relied upon in annual subscriptions. The committee would therefore like to see a great addition to the list of annual and life subscriptions, and earnestly appeal for Christmas and New Year Gifts. Secretary, Mr. G. Scudamore.

**The Hospital for Women,** Soho Square, W.—This institution (founded in the year 1842) claims the distinction of being the first established in this or any other country, exclusively for the treatment of women's diseases. There are 61 beds in constant use, and, as the hospital possesses no endowment, funds for their maintenance are much needed. The committee very earnestly appeal for additional annual subscriptions not only to Londoners, but to the whole of the United Kingdom, from all parts of which patients are received. Secretary, Mr. David Cannon.

MISCELLANEOUS—SPECIAL.

**Central London Ophthalmic Hospital,** Gray's Inn Road, W.C.—This hospital, which has treated over 300,000 patients during the fifty-three years of its existence, is in much need of funds. Secretary, Mr. J. G. Bryant.

**Dental Hospital of London,** Leicester Square, W.C.—The state of affairs set forth in our Christmas number last year has but little changed, and funds for building the new hospital are still urgently needed. Secretary, Mr. J. Francis Pink.

**London Lock Hospital and Home,** Harrow Road, W.—Rescue work, as well as the cure of the patients who present themselves for admission, is the aim of this institution, and the good work has only to be known to meet with the support it deserves. Funds are much wanted to meet the deficiency in income, owing to the falling off in donations, and a heavy expenditure on sanitary and other work. Secretary, Mr. A. W. Cruikshank.

**London Fever Hospital,** Liverpool Road, Islington, N.—The institution is dependent upon voluntary support, and donations and subscriptions will be gratefully received, especially as the alterations and additions to the hospital, now being carried out, and the building of a convalescent home, are taxing to the utmost the resources of the institution. Secretary, Major W. Christie.

**National Orthopædic Hospital,** 324, Great Portland Street, W.—The hospital's pressing needs are first to see the end of a debt of £1,300 on the building, and then to increase its list of annual subscribers to three times its present amount. Secretary, Mr. H. J. Tresidder.

**Royal Eye Hospital,** St. George's Circus, Southwark, S.E.—The work of this hospital increases year by year, and in 1896 16,228 cases were treated. Secretary, Mrs. J. E. Cope.

**Royal London Ophthalmic Hospital,** Moorfields, E.C.—An urgent appeal is made by the board of management for funds in support of this institution. The patients numbered in 1896, in-patients, 2,006; out-patients, 26,074. All patients are admitted free, and without letters of recommendation. Secretary, Mr. Robert J. Newstead.

**Royal Orthopædic Hospital,** 297, Oxford Street, W.—The majority of out-patients at this hospital come, as might be expected, from the metropolitan districts, but of the 155 in-patients admitted in 1896, 88, or 57 per cent. came from the provinces, including Ireland and the Channel Islands. Secretary, Mr. Tate S. Mansford.

**St. Luke's Hospital,** London, E.C.—The committee have continued to carry out the much-needed and extensive repairs to the hospital necessitated by the age of the building (erected 1787), and these works must be continued for some time to come, seriously drawing on the funds, which are very urgently in need of support. The charity has been treating insanity for 150 years; is now devoted solely to patients drawn from the "middle class" population whose means are limited, and for whom the State provides no adequate relief. Secretary, Mr. W. H. Baird.

**Western Ophthalmic Hospital,** 155, Marylebone Road, W.—At present existing in a private house, quite unfitted for hospital purposes, and growing needs, an appeal for funds for building a new hospital is being made. Help is earnestly asked for. Secretary, Mr. A. H. E. Grahame.

### GENERAL CHARITIES.

**Association for the Oral Instruction of the Deaf and Dumb.**—This association was the first to publicly introduce into the United Kingdom the German or pure oral system for teaching deaf and so-called dumb children to speak *viva voce*, and to understand the spoken words of others by lip-reading. The expenses, which are very heavy, are met by voluntary contributions and fees, which fall short of what is needed by about £700 per annum. Financial aid is most earnestly solicited, and cheques may be directed to the Treasurer or the Director at the offices, 11, Fitzroy Square, W.

**Bethnal Green Free Library.**—During the present year a new Free Lending Department has been opened to meet the demands of the inhabitants, which has met with great success. The poverty in the surrounding neighbourhood compels the committee to make an urgent appeal for funds to meet outstanding liabilities. Contributions will be thankfully received by the Librarian, at the Bethnal Green Free Library, E., or by the Treasurer, Mr. F. A. Bevan, 54, Lombard Street, E.C.

**British Home for Incurables,** Crown Lane, Streatham Common, S.W. Instituted in 1861.—This home is intended for those who, having once been in a position of comfort, are now in necessitous circumstances, and are either bedridden or greatly dependent on others in the various offices of life. For these the institution provides medical attendance, nursing, and the comforts of a home for life. Annuities of £20 are conferred upon such as are incurable, but not wholly destitute, in order that they may continue to reside with friends or relatives, who may be able to render them some further assistance. The charity extends its benefits to all parts of the United Kingdom. Since the foundation of the charity 919 candidates have been elected, viz., 236 in-patients, and 683 pensioners with annuities of £20 each. Secretary, R. G. Salmond, 72, Cheapside, E C.

**City of London Truss Society,** 35, Finsbury Square, E.C.—At the present time about 10,000 of both sexes and all ages are treated annually. The great growth in the relief afforded by the society in recent years is shown by the fact that of the 520,000 patients treated during the ninety years of its existence nearly one-half have been relieved within the last twenty-five years. Secretary, Mr. John Whittington.

**Irish Distressed Ladies' Fund,** 17, North Audley Street, W.—Relief is given independently of any question of politics or religion; employment is found, when possible, for those able to work; pecuniary help is given to the aged and infirm; and the education of children is paid for.

**London Orphan Asylum,** Watford.—During the past year the managers have admitted the unprecedented number of 113 children to the benefits of the institution, as a mark of their gratitude for the support bestowed through the medium of the institution upon the widow and the fatherless during the whole of Her Majesty's Record Reign. One interesting feature of the work done by the managers is that they appeal for help to enable them to assist cases which would be precluded by age from standing at subsequent elections, and that by this means all who make any reasonable effort ultimately secure election, only 3 per cent. having known failure within the past four years. £3,000 has had to be borrowed from the bankers. Secretary, Mr. Henry C. Armiger, 21, Great St. Helen's, E.C.

**London Schools Dinner Association.**—Established in 1889, to provide cheap or free meals for necessitous children attending the public elementary schools of London, this association is doing an excellent work. The annual income now averages about £1,500, but it is reckoned that double that sum is necessary to meet the needs of the children. Secretary, Mr. T. A. Spalding, 37, Norfolk Street, Strand, W.C.

**London Society for Teaching the Blind,** 10, Upper Avenue Road, Hampstead Road, N.W.—This society educates children, blind and partially blind, in general and technical instruction, viz., music, piano tuning, basket-making, needlework, &c. Both sexes and all denominations received. The cost of carrying out this work is £2,800 a year, and the income of this society falls short of this amount by £600. Secretary, Captain G. G. Webber, R.N.

**Mary Wardell Convalescent Home for Scarlet Fever,** Stanmore, Middlesex.—The only home for convalescents from scarlet fever. The demand on its 40 beds is great, and expenses are heavy. Funds are greatly needed to meet the yearly expenses, and the debt unavoidably incurred a year or so ago in renovating the home, has not yet been liquidated. Subscriptions to Miss Mary Wardell.

**Orphan Working School,** Haverstock Hill, N.W., and Hornsey Rise, N. Offices, 73, Cheapside, E.C.—This is a national and undenominational institution which maintains 500 children, varying in age from infancy to fourteen or (in special cases) fifteen years. It is greatly in want of funds at the present time, urgently needed to meet immediate and pressing liabilities. This orphanage is the oldest one of the kind in the metropolis, and a large percentage of scholars turn out satisfactorily. Secretary, Mr. Algernon C. P. Coote, M.A.

**Royal Albert Orphan Asylum,** Bagshot.—This institution affords a home and industrial training for about 200 fatherless children. No canvassing or sale of votes is permitted. Help is urgently needed, as the debts are heavy, and a curtailment of the work will be necessary unless funds are forthcoming. Secretary, Mr. H. W. Tatum. Office, 62, King William Street, E.C.

**Royal Blind Pension Society,** 235, Southwark Bridge Road, S.E.—This society provides pensions, by monthly instalments, for 297 blind people. Further contributions, particularly annual subscriptions, are required to enable it to give aid to 200 candidates who are approved as eligible for charitable assistance. Secretary, Mr. W. E. Terry.

**Royal Sea Bathing Infirmary,** founded at Margate 1791.—This institution has been so badly supported that for some years past many of the wards have been closed. The efforts to remedy this state of affairs have recently been fairly successful, and by the end of this year it is hoped that only 70 of the 220 beds will be unavailable for patients. Secretary, Mr. A. Peirce, 30, Charing Cross, S.W.

**Surgical Aid Society,** Salisbury Square, E.C.—The net expenditure for the year amounted to £12,750, of which 84 per cent. was for actual relief. During the past year the large total of 22,247 appliances were given away. There is ample scope for very considerable extension of these benefits, and, therefore, the committee earnestly appeal for contributions. Secretary, Mr. Richard C. Tresidder.

## Figures and Tables

**Figure f1:**
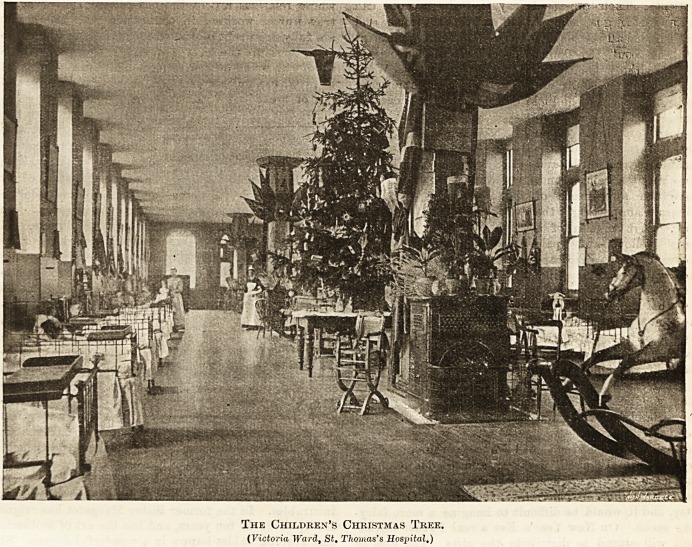


**Figure f2:**
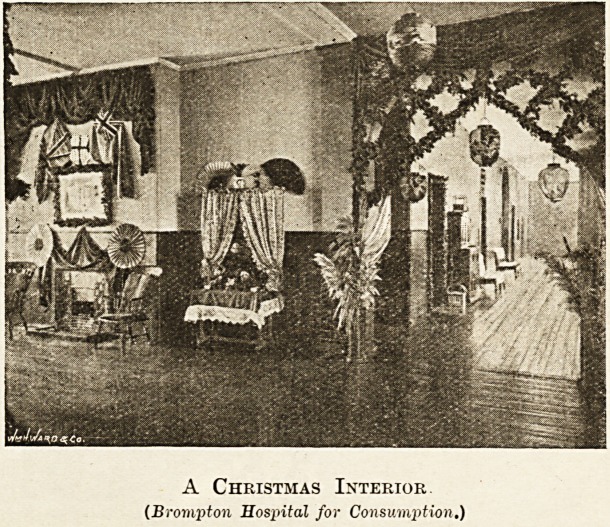


**Figure f3:**
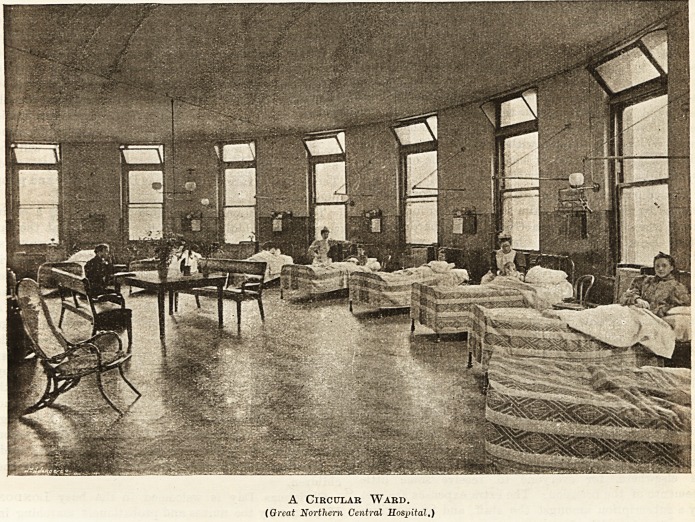


**Figure f4:**
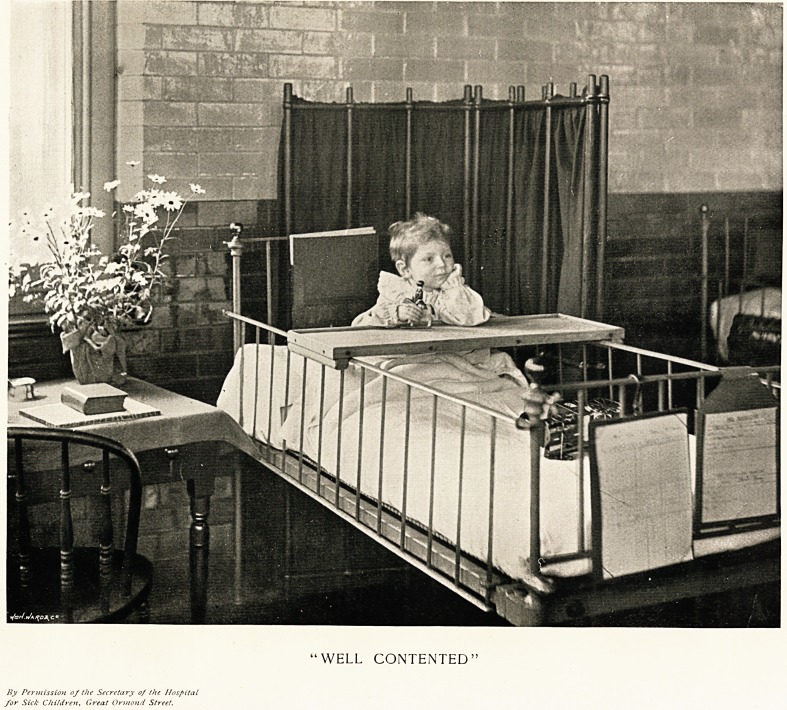


**Figure f5:**
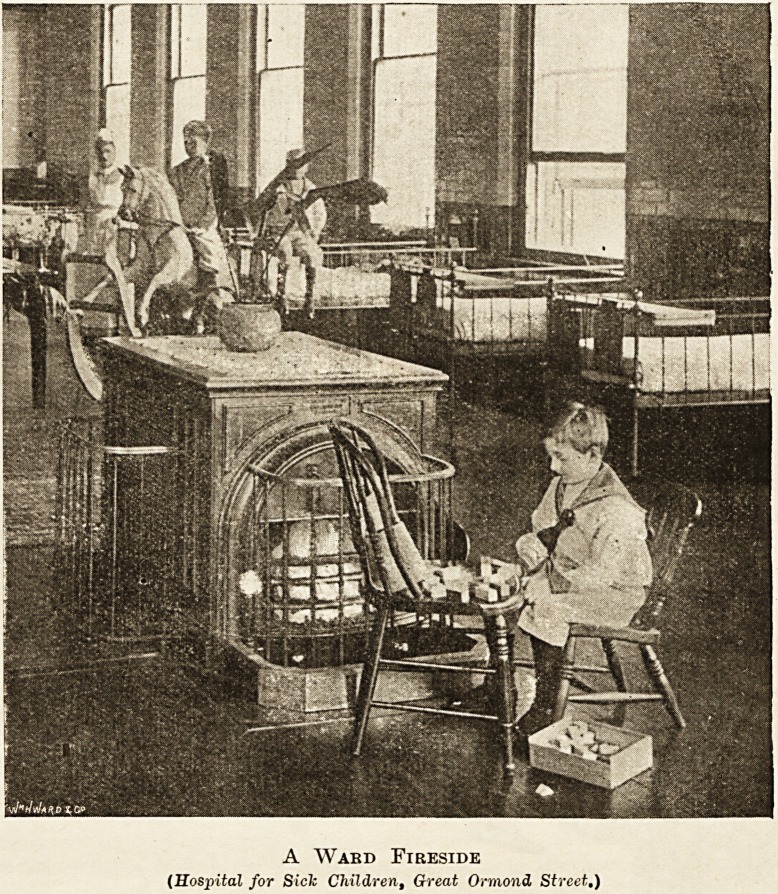


**Figure f6:**